# Serotype distribution and antibiogram of *Streptococcus parauberis* isolated from fish in South Korea

**DOI:** 10.1128/spectrum.04400-22

**Published:** 2023-08-09

**Authors:** Yoonhang Lee, Nameun Kim, HeyongJin Roh, Diem Tho Ho, Jiyeon Park, Ju Yeop Lee, Yoon-Jae Kim, Hyo-Young Kang, Jungmin Lee, Jun-Young Song, Ahran Kim, Myoung Sug Kim, Miyoung Cho, Hye Sung Choi, Chan-Il Park, Do-Hyung Kim

**Affiliations:** 1 Department of Aquatic Life Medicine, Pukyong National University, Busan, South Korea; 2 Pathology Research Division, National Institute of Fisheries Science, Busan, South Korea; 3 Department of Marine Biology and Aquaculture, Gyeongsang National University, Tongyeong, South Korea; USGS, Eastern Ecological Science Center, Kearneysville, West Virginia, USA

**Keywords:** *Streptococcus parauberis*, serotype, antimicrobial susceptibility, antimicrobial resistance gene

## Abstract

*Streptococcus parauberis* is the dominant etiological agent of streptococcosis, the most devastating bacterial disease in the olive flounder farming industry in South Korea. In this study, the distribution of serotypes, antimicrobial susceptibility, and presence of antimicrobial resistance genes (ARGs) in *S. parauberis* isolates obtained between 1999 and 2021 was thoroughly investigated to gain insight into the dynamics of their presence and the relationship between serotypes and antimicrobial resistance. Disk diffusion testing of 103 isolates against 10 antimicrobial agents was performed, and epidemiological cut-off values generated through normalized resistance interpretation analysis were used to classify wild-type (WT) and non-wild-type (NWT) populations. Principal component analysis and hierarchical clustering were implemented to achieve an understanding on the relationship between serotypes and antimicrobial resistance patterns. PCR-based serotyping showed that serotype Ia (67.1%) was the most prevalent in South Korea, followed by serotypes Ib/Ic (25.2%) and II (7.7%). The highest proportion of isolates was assigned to NWT against amoxicillin (80.6%), followed by oxytetracycline (77.7%) and erythromycin (48.5%). The time-scale data showed that recently obtained serotypes Ib/Ic and II isolates tended to be categorized as NWT populations resistant to more antibiotics, possibly due to microbial adaptation to antibiotic pressure. ARGs responsible for resistance to oxytetracycline and erythromycin were found only in NWT populations in serotype Ia [*tet*(S) and *erm*(B), respectively], and serotype II [*tet*(M) and *mef*(J)-*msr*(I), respectively]. We also found that the *mef-msr* gene pair in *S. parauberis* serotype II might be involved in low-level resistance to erythromycin.

**IMPORTANCE:**

This study presents serotype distribution and antimicrobial susceptibility data along with the antimicrobial resistance genes (ARGs) of *Streptococcus parauberis*, which is an important bacterial fish pathogen worldwide. In particular, almost all oxytetracycline and erythromycin non-wild-type (NWT) populations harbored *tet*(S) or *tet*(M), and *erm*(B) or *mef*(J)-*msr*(I), respectively. Interestingly, these ARGs were distributed in a highly serotype-dependent manner, resulting in a clear correlation between the antibiogram and serotype distribution. Moreover, recent isolates belonging to serotypes Ib/Ic and II tended to be more frequently categorized as NWT against antimicrobials, including amoxicillin and cefalexin compared to old isolates, while a dramatic decrease in erythromycin and clindamycin NWT frequencies was observed in recent serotype Ia isolates, which lacked *erm*(B). These variations might be attributed to shifts in the antibiotics employed in South Korean aquaculture over time. The overall findings would provide important background knowledge for understanding the epidemiology of *S. parauberis* infection in aquaculture.

## INTRODUCTION

Streptococcosis is one of the most important bacterial diseases in aquaculture worldwide, causing severe mortality in various fish species, including the olive flounder (*Paralichthys olivaceus*), starry flounder (*Platichthys stellatus*), turbot (*Scophthalmus maximus*), and striped bass (*Morone saxatilis*) (1 – 4). Since the early 2010s, *Streptococcus parauberis* has been a dominant causative agent of streptococcosis in farmed olive flounder in South Korea (5). Commercial formalin-killed cell vaccines and the use of antibiotics are options for the prevention and treatment of *S. parauberis* infections. However, *S. parauberis* infections still occur in the field (6). This might be due to the presence of different serotypes and the emergence of antibiotic-resistant strains.

Currently, several serotypes of *S. parauberis* (i.e., Ia, Ib, Ic, II, III, and IV) have been proposed, based on differences in the sequences of their capsular polysaccharide (CPS) genes (4, 7, 8). Tu et al. (9) developed a multiplex PCR assay to identify three serotypes (Ia, Ib/Ic, and II) of *S. parauberis* based on the sequence of the *wzy* gene encoding CPS polymerase, indicating that serotyping can be performed by PCR, as an alternative method to traditional serotyping using anti-sera. Previously, serotypes I and II were isolated from farmed olive flounder in South Korea and Japan (8, 10). Serotype III was isolated from turbot in Spain (4) and striped bass in the United States (2), and a single bovine isolate was assigned to serotype IV (11). In general, candidate vaccine strains are selected by taking into account the genotypes and serotypes currently circulating in the region (12). The selection of appropriate vaccine strains is an important element in implementing vaccination policies for the control of diseases (e.g., dengue and influenza) (13).

The emergence of drug-resistant bacteria under antibiotic selection pressure in aquaculture has become more evident and is a significant threat worldwide (14). Antimicrobial resistance can spread to microbial communities through the horizontal gene transfer of antimicrobial resistance genes (ARGs), which are often found in mobile genetic elements such as plasmids, prophages, and transposons (15). Previous studies (16 – 18) found that several *S. parauberis* isolates harbored *tet*(S), *tet*(M), or *erm*(B), exhibiting resistance to oxytetracycline or erythromycin. However, those studies applied clinical breakpoints as described in Clinical and Laboratory Standards Institute (CLSI) M31-A3 guidelines (19) developed for microbes derived from terrestrial animals, to discriminate resistance from susceptibility. For example, Woo et al. (18) reported that 18 erythromycin-susceptible isolates categorized by the breakpoint (19) harbored *erm*(B), indicating that incorrect interpretive criteria were used in the study and therefore not suitable for precise discrimination of antimicrobial resistance in *S. parauberis*. Instead, an epidemiological cut-off value (ECV) can be established based on arrays of antimicrobial susceptibility test results and applied as an interpretative criterion to categorize a bacterial population into two groups: those that are fully susceptible (wild type, WT), and those that exhibit reduced susceptibility by acquired mechanism of resistance (non-wild type, NWT) (20). Unlike the clinical breakpoint, the ECV is generated without consideration of pharmacokinetics/pharmacodynamics and results obtained from clinical trials. Therefore, the ECV should not be used as a predictor of clinical success but as epidemiological surveillance tool for bacterial antimicrobial resistance (20). Although we previously established ECVs for *S. parauberis* based on disk diffusion data using 75 isolates (21), serotyping and the detection of ARGs were not performed. This study used a total of 103 isolates, including recently isolated strains.

The relationship between serotype and antimicrobial resistance has been reported for *S. parauberis* (17, 22), as well as other streptococcal species (23 – 25). Studies (17, 22) using *S. parauberis* isolates from *P. olivaceus* between 2002 and 2008 showed that *tet*(M) was present in only 32 serotype II isolates and absent in 44 serotype I isolates. Meng et al. (17) found that *S. parauberis* serotype II isolates commonly exhibited intermediate resistance to erythromycin but were not able to specify any relevant ARGs. In *Streptococcus agalactiae*, macrolide-resistance genes were distributed with a clear correlation to serotypes: *mef*(A) and *erm*(A) were most common in serotypes Ia (55%) and Ib (75%), respectively, whereas *erm*(B) was most prevalent in both serotype III (73%) and V isolates (75%) (23). *Streptococcus pneumoniae* serotype 19A, a non-vaccine type, has become the most prevalent serotype worldwide since the implementation of the seven-valent pneumococcal conjugate vaccine (26). Previous studies have demonstrated that serotype 19A could frequently acquire and exhibit antimicrobial resistance to multiple drugs under antibiotic pressures, leading to its competitive survival and prevalence in multiple countries (24, 25). The overall findings from previous studies indicate that the surveillance of serotype distribution along with antimicrobial resistance could facilitate a better understanding of the epidemiology of *S. parauberis* infections in fish.

In this study, ECVs were estimated based on antibiotic disk diffusion data that were generated using 103 *S*. *parauberis* isolates collected from 1999 to 2021 and used to categorize WT and NWT populations of *S. parauberis*. Serotyping and ARG detection were also conducted to gain insight into the dynamics of serotype prevalence and their association with antimicrobial resistance patterns.

## RESULTS

### Serotyping of South Korean *S. parauberis* isolates

All bacterial isolates (*n* = 103) used in this study were identified as *S. parauberis*, and only three serotypes, Ia, Ib/Ic, and II, were found, as shown in Table S1. Of the 103 isolates, 75, 20, and 8 strains were isolated from flounder farms in Jeju Island, the Gyeongsang province, and the Jeolla province in South Korea, respectively (Table S1). Also, the majority of isolates were derived from olive flounder (98 out of 103 strains), while only five strains were from starry flounder. The most prevalent serotype in the South Korean *S. parauberis* isolates used in this study was Ia at ~67% (69 out of 103 strains), followed by Ib/Ic (~25%) and II (~8%) (Table 1). Serotype Ia was the most frequently isolated every year, except for 2005, when serotype Ib/Ic was the most dominant (93%). Serotype Ia was the predominant isolate in Jeju Island and the Gyeongsang province, while all isolates from the Jeolla province were serotype Ib/Ic.

**TABLE 1:** Serotyping of 103 *Streptococcus parauberis* strains isolated in South Korea

Year of isolation	Proportion of serotype		
	Ia	Ib/Ic	II
1999	100% (4/4)	0% (0/4)	0% (0/4)
2003	50% (2/4)	0% (0/4)	50% (2/4)
2004	50% (3/6)	33% (2/6)	17% (1/6)
2005	0% (0/15)	93% (14/15)	7% (1/15)
2007	100% (2/2)	0% (0/2)	0% (0/2)
2013	100% (3/3)	0% (0/3)	0% (0/3)
2014	83% (5/6)	17% (1/6)	0% (0/6)
2015	100% (5/5)	0% (0/5)	0% (0/5)
2018	73% (11/15)	27% (4/15)	0% (0/15)
2019	75% (12/16)	25% (4/16)	0% (0/16)
2021	81% (22/27)	4% (1/27)	15% (4/27)
Total	67% (69/103)	25% (26/103)	8% (8/103)

### Analysis of zone diameters of *S. parauberis* using normalized resistance interpretation

The distribution of inhibitory zone sizes obtained from the disk diffusion testing of 103 *S*. *parauberis* isolates against 10 antimicrobial agents is shown in Table S2. The inhibitory zone diameters of the two reference strains were within the normal ranges in accordance with the CLSI guidelines (27). The histograms and ECVs of the respective antibiotics determined through normalized resistance interpretation (NRI) analysis are shown in Fig. S1 and Table 2. Isolates exhibiting inhibitory disk zone size ≥ECVs were designated the WT population since they fit the definition of the lower limit of the isolates lacking resistance mechanisms. Abbreviations for the antibiotic agents are summarized in Table 2. Overall, approximately 81% of the *S. parauberis* isolates used in this study were classified as NWT with resistance against AMX, followed by OTC (~77%) and ERY (~48%). In contrast, only ~6%, ~3%, 0%, and 0% of the *S. parauberis* isolates were categorized as NWT with resistance against ENR, FFC, CHL, and SXT, respectively.

**TABLE 2:** Summary of results of normalized resistance interpretation analysis

Antibiotics	ECV (mm)^*a*^	SD (mm)^*b*^	WT (%)^*c*^
Amoxicillin (AMX)	34	2.3	19.4
Cephalexin (CFL)	23	4.6	83.8
Ceftiofur (EFT)	29	2.9	72.8
Oxytetracycline (OTC)	26	2.5	23.3
Erythromycin (ERY)	29	2.6	52.4
Clindamycin (CLI)	23	3.2	69.9
Chloramphenicol (CHL)	22	3.1	100
Florfenicol (FFC)	25	2.4	97.1
Enrofloxacin (ENR)	18	3.5	100
Sulfamethoxazole/trimethoprim (SXT)	20	2.2	94.2

^*a*^ ECV, epidemiological cut-off value.

^*b*^ SD, standard deviation.

^*c*^ WT, wild type.

**Fig 1:**
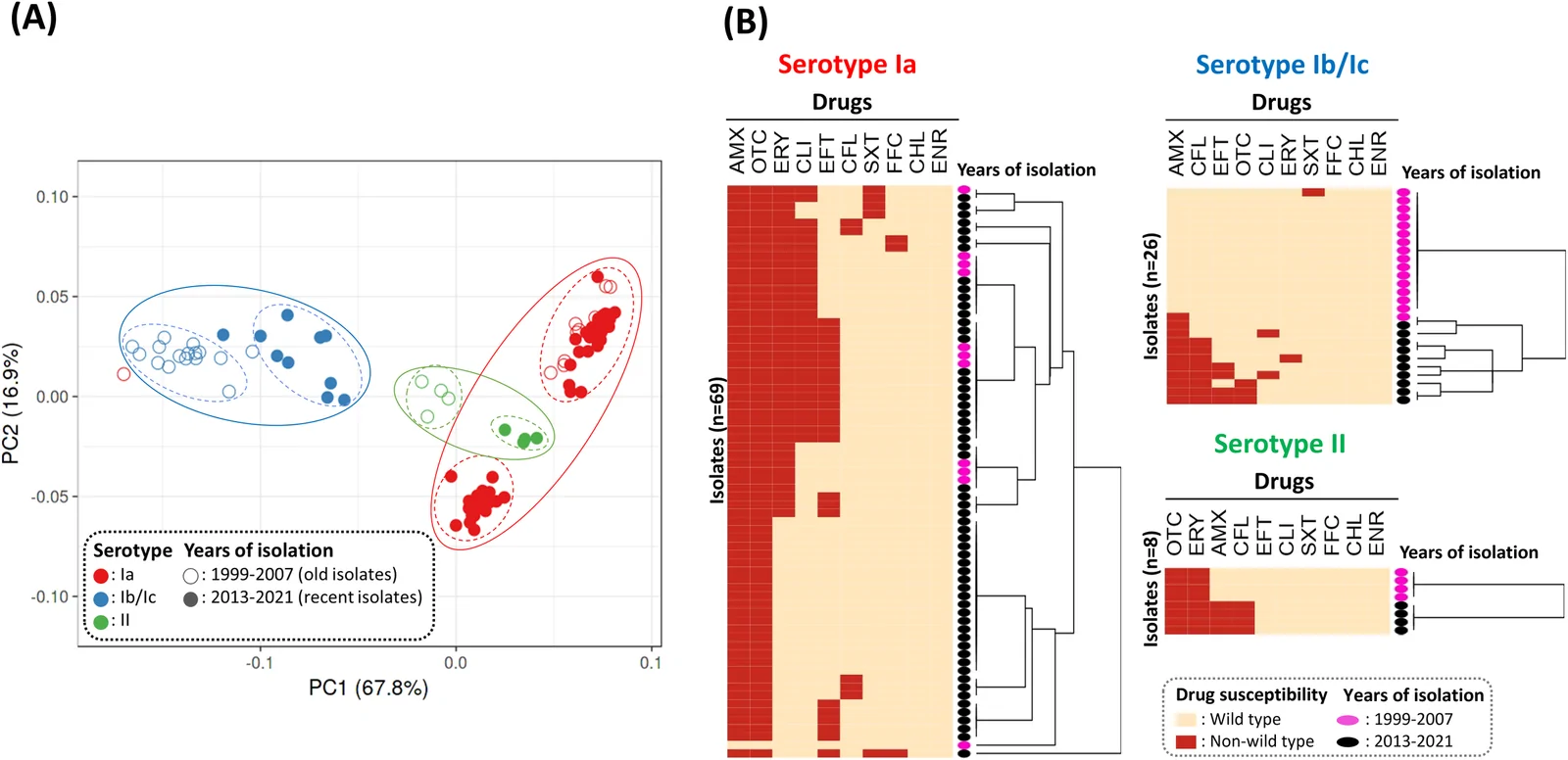
Principal component analysis (PCA; **A**) and hierarchical clustering analysis (**B**) based on the antibiogram of 103 *Streptococcus parauberis* isolates against 10 antibiotics. (**A**) The PCA plot captures the variance in the data set composed of normalized inhibitory zone diameters obtained from the disk diffusion tests of 103 *S*. *parauberis* strains. The *X* and *Y*-axes show principal components 1 and 2, which explain 67.8% and 16.9% of the total variance, respectively. The colors of the circles represent the serotype. Open and closed circles indicate the isolated year, categorized as 1999–2007 and 2013–2021, respectively. (**B**) Heatmaps show the wild-type (beige) and non-wild-type (red) distributions of *S. parauberis* isolates according to serotype. The black and pink ellipses located on the right side of the heatmap indicate the year of isolation, categorized as 1999–2007 and 2013–2021, respectively. Abbreviations for the antibiotic agents are shown in Table 2.

**Fig 2:**
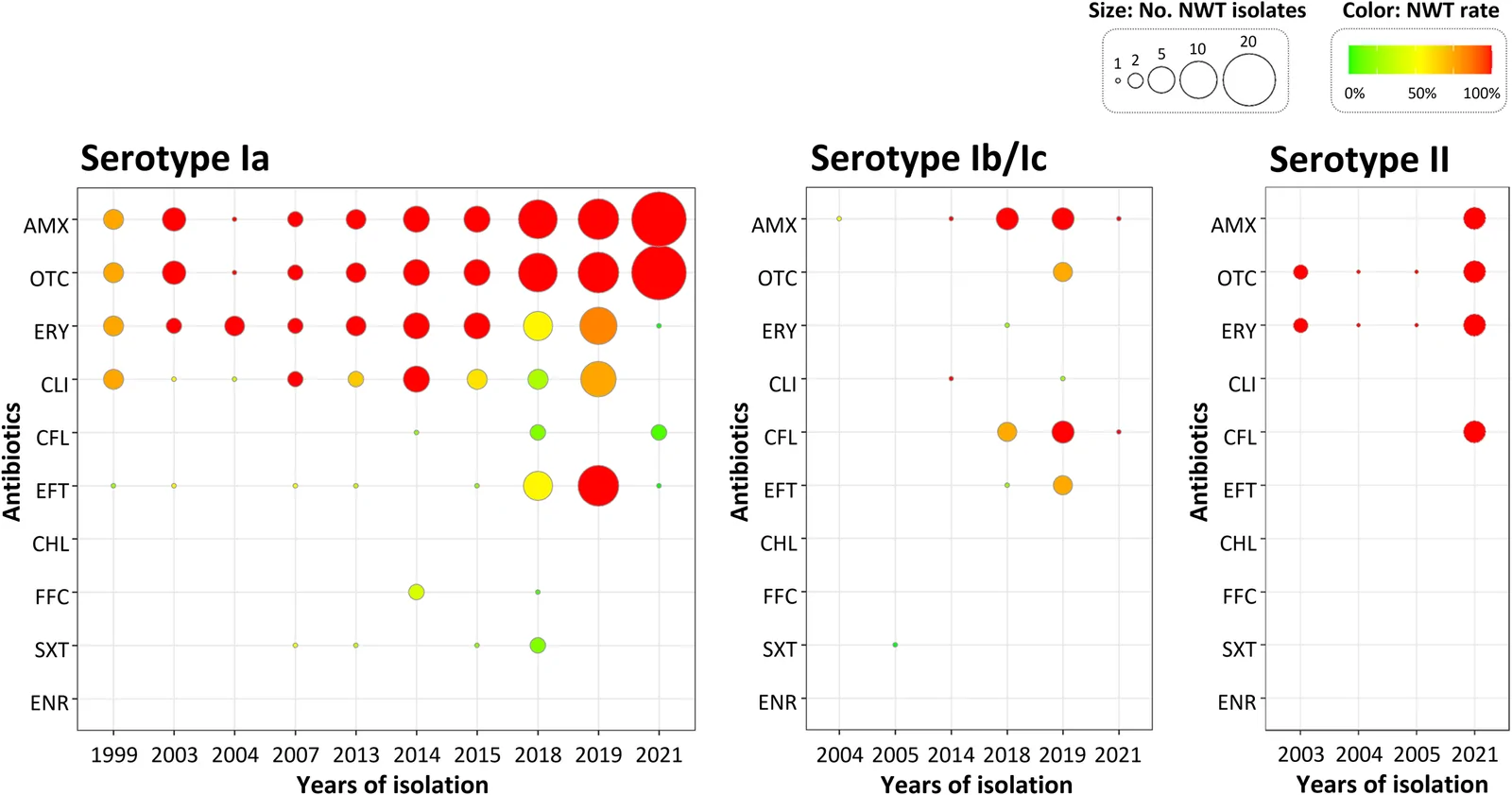
Year-wide distribution of non-wild-type (NWT) populations (%) of three serotypes of *Streptococcus parauberis* isolates against 10 antibiotics. The color of the dots indicates the NWT proportion, while the size indicates the number of NWT populations in the respective isolation year. Abbreviations for the antibiotic agents are shown in Table 2.

Based on principal component analysis (PCA) and hierarchical clustering analysis, the majority of isolates, with few exceptions, were significantly clustered depending on their serotype, and partially the year of isolation (Fig. 1). Based on the region of isolation, strains isolated from the Gyeongsang and Jeolla provinces could be grouped separately, while isolates from Jeju Island exhibited relatively more diverse antimicrobial susceptibility patterns (Fig. S2). Almost all isolates belonging to serotypes Ia and II were categorized as NWT with resistance against OTC and AMX, and OTC and ERY, respectively, indicating a possible decrease in the therapeutic effects of those antimicrobials (Fig. 1B). The median number of antibiotics to which the isolates exhibit NWT for serotype Ib/Ic was comparatively lower than that of the other two serotypes (Fig. S3). Interestingly, the recent isolates belonging to serotypes Ib/Ic and II were categorized as NWT with resistance against more antimicrobials (Fig. 2). Recent serotype Ib/Ic isolates (*n* = 10) were classified as NWT with resistance to seven tested antibiotics including AMX (100%) and CFL (80%), while old isolates (*n* = 16) were WT to most of the tested drugs (Fig. 1B and 2). However, in serotype Ia, a dramatic decrease in NWT frequency with resistance against ERY and CLI was observed from ~96% and ~71% in 1999–2015 to ~38% and ~27% in 2018–2021, respectively. In the case of EFT, the proportion of NWT in serotype Ia isolates was ~55% (6/11) in 2018 and 100% (12/12) in 2019, but dropped significantly to ~5% (1/21) in 2021 (Fig. S3). This might be linked to the recent introduction of injectable EFT in South Korean aquaculture, which has been released since 2018 (28).

### Identification and detection of ARGs in *S. parauberis*

**Fig 3:**
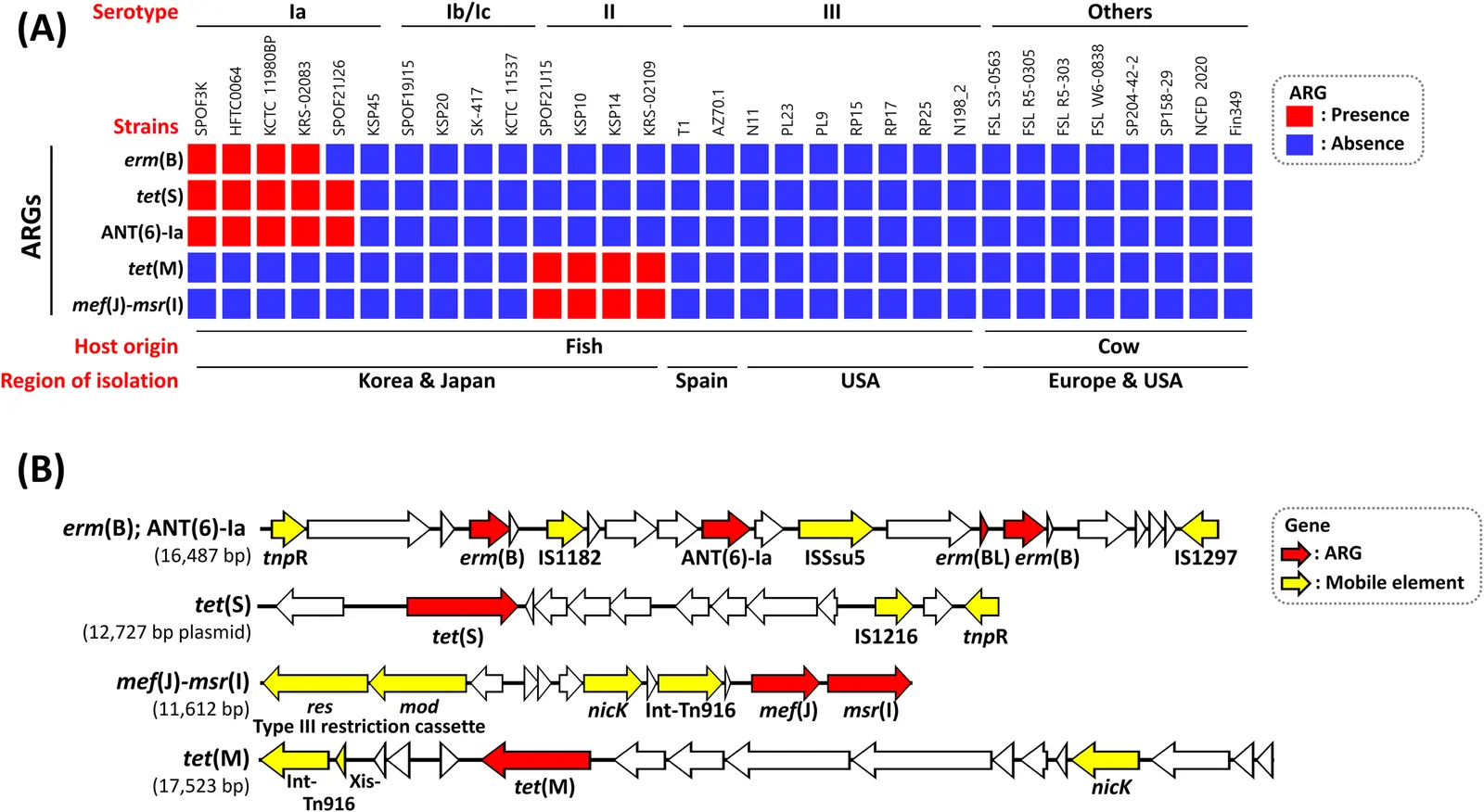
Distribution of antibiotic resistance genes (ARGs) in 31 *Streptococcus parauberis* genome sequences. (**A**) The heatmap presents the presence and absence of ARGs in each isolate, as shown in the red and blue boxes, respectively. (**B**) Gene organization of four ARG-carrying mobile element structures in *S. parauberis* serotypes Ia and II. The direction of the arrow indicates the direction of transcription. ARGs and mobile element-related genes are shown in red and yellow arrows.

**Fig 4:**
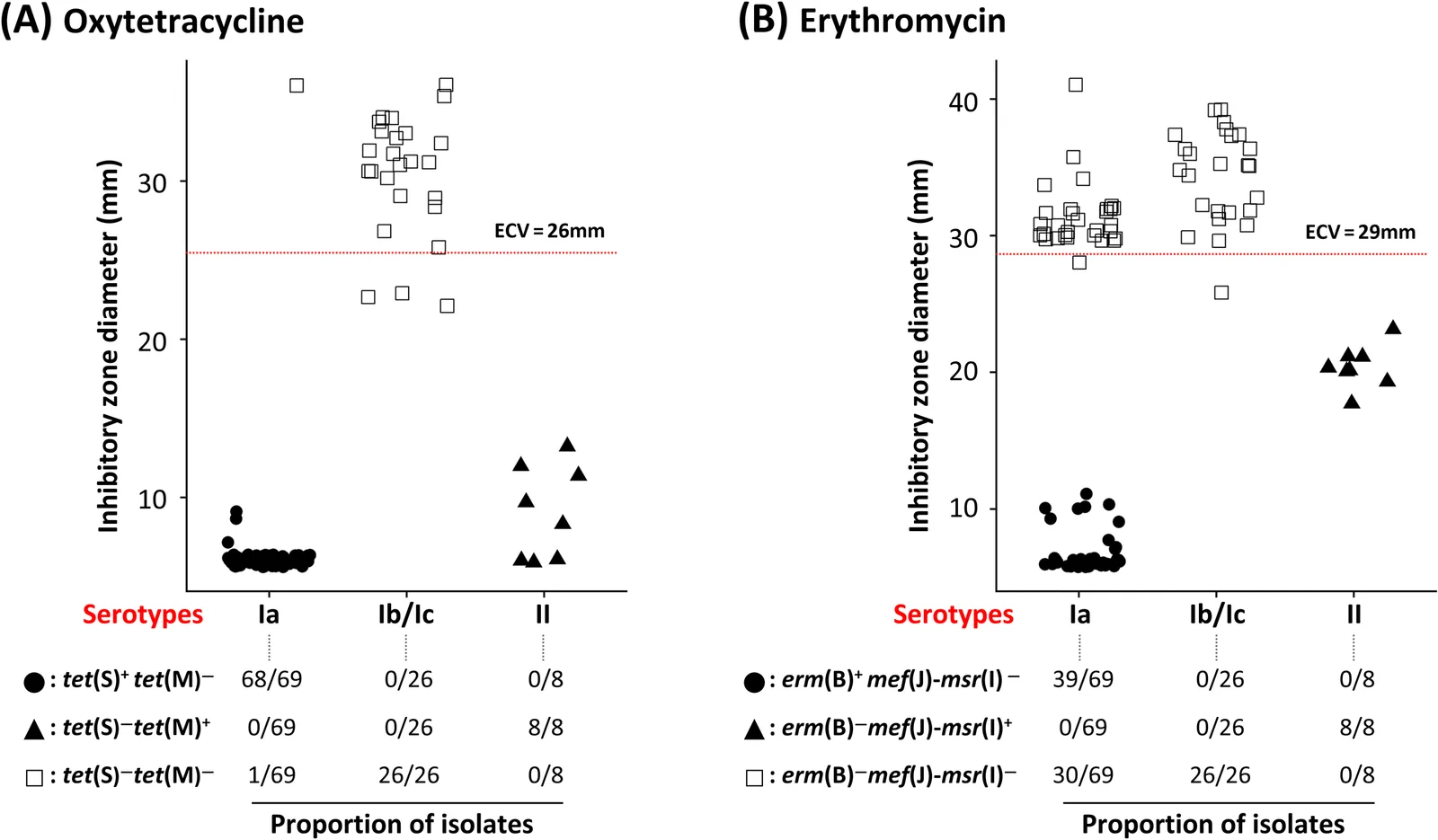
Distribution of inhibitory zone diameters of 103 *Streptococcus parauberis* isolates to oxytetracycline (**A**) and erythromycin (**B**) shown with the proportion of isolates harboring antimicrobial-resistance genes. Inhibitory zone diameters of 103 *S*. *parauberis* isolates are shown in filled circles [*tet*(S)^+^ *tet*(M)^−^ and *erm*(B)^+^ *mef*(J)-*msr*(I)^−^ in panels A and B, respectively], filled triangles [*tet*(S)*^−^tet*(M)^+^ and *erm*(B)^−^ *mef*(J)-*msr*(I)^+^ in panels A and B, respectively], and empty squares [*tet*(S)^−^ *tet*(M)^−^ and *erm*(B)^−^ *mef*(J)-*msr*(I)^−^ in panels A and B, respectively]. The horizontal red lines indicate the epidemiological cut-off values (ECVs). The proportion of respective genotypes in every serotype is shown below the scatter plots.

This study identified six ARGs from pan-genomic analysis using 31 *S*. *parauberis* genomes including four sequenced genomes in the current study (KSP45, SPOF19J15, SPOF21J2, and SPOF21J26), as shown in Fig. 3. Information on whole-genome sequences used in this study are available in the supplementary results and Table S4. Three ARGs, *erm*(B), *tet*(S), and ANT (6)-Ia, were found only in serotype Ia genomes, whereas *tet*(M) and *mef*(J)-*msr*(I) were present only in serotype II (Fig. 3A). *tet*(S) was present in a ~12 kb-sized plasmid, while two paralogous copies of *erm*(B) [with or without a leader peptide-encoding gene, *erm*(BL)] and ANT (6)-Ia were contained in the chromosome, as illustrated in Fig. 3B. Unlike other serotype Ia strains, the *tet*(S)-containing plasmid was exceptionally absent in the genome of KSP45, which exhibited WT to OTC (Fig. 3A). The transposon-mediated gene cluster carrying *erm*(B) could not be identified in genome sequence of KSP45 and SPOF21J26, which were WT to ERY (Fig. 3A). Consistent with this, among the serotype Ia isolates used in this study, NWT isolates with resistance to OTC and ERY harbored *tet*(S) (*n* = 68/69) and *erm*(B) (*n* = 39/69), respectively (Fig. 4A). All eight serotype II isolates used in this study were classified as NWT with resistance against OTC and ERY and harbored *tet*(M) and *mef*(J)-*msr*(I) (Fig. 4). Based on the inhibitory zones generated by serotype II isolates against ERY, the *mef*(J)-*msr*(I) genes might be responsible for the relatively low-level resistance to ERY (Fig. 4B). Although the ARGs mentioned above were not found in serotype Ib/Ic isolates in this study, three and one isolates were categorized as NWT with resistance against OTC and ERY, respectively. However, the inhibitory zone diameters of these isolates were distributed close to ECVs, unlike the other ARG-carrying isolates.

## DISCUSSION

This study aimed to track the trends of the antibiotic-resistance patterns of *S. parauberis* isolated between 1999 and 2021, along with serotyping. In fact, we used 31 whole-genome sequencing of *S. parauberis,* including nine genomes sequenced in our laboratory, and compared our results with as relevant many references showing antimicrobial susceptibility and serotypes of the pathogen as possible (4, 7, 8, 10, 16 – 18, 21, 22, 29, 30) to overcome the limited numbers of strains (*n* = 103) used. We found profound diversity in terms of antibiotic resistance in *S. parauberis* through the establishment of ECVs for 10 antimicrobial agents based on disk diffusion test data using 103 isolates (Fig. 1). The number of WT isolates in the respective, tested antibiotics were diverse (*n* = 20–103), which satisfied the requirement of at least 20–30 observations from fully susceptible isolates in NRI analysis (31). In NRI analysis, standard deviations for normalized WT inhibitory zone sizes are calculated, thereby providing quantitative measure of the precision of the antimicrobial susceptibility data sets (31). The standard deviations of 9 out of the 10 tested antibiotics satisfied the criterion of ≤3.95 mm (31), except for CFL (4.3 mm) (Table 2). Based on the ECVs for OTC and ERY, with just a few exceptions, only NWT populations of *S. parauberis* harbored the respective ARGs, as shown in Fig. 4. This indicates that most ECVs obtained in this study were accurate and could be utilized as local criteria for the discrimination of WT from NWT *S. parauberis* isolates.

Based on similar but independent studies (10, 18), it can be concluded that serotype Ia has been the most dominant serotype in South Korea since the late 1990s, followed by serotypes Ib/Ic and II (Table 1). Since the appearance of approximately a dozen serotype Ib/Ic isolates collected in 2005, they have decreased, which is in partial agreement with the results of Kim et al. (10), showing that serotype Ib/Ic was the most prevalent serotype in the period of 2005–2009. Kim et al. (10) also pointed out that the isolation rate of serotype II was extremely low in the 2010s, but started to increase after 2019. In this study, it was found that serotypes Ia and Ib/Ic were predominant in the Gyeongsang (19 out of 20 isolates) and Jeolla provinces (8 out of 8 isolates), respectively (Table S1), which were also distinct in terms of their antibiotic susceptibility patterns (Fig. S2). However, as these isolates were clustered with the isolates of respective serotypes from other geographical sources based on the antibiogram (Fig. S2), it seems highly likely that the region-specific antimicrobial susceptibility patterns in the Gyeongsang and Jeolla provinces were substantially affected by the regional predominance of serotypes. Along with previous studies (17, 18), data from this study suggest that the existence of ARGs in *S. parauberis* has been significantly conserved in a serotype-dependent manner (Fig. 4). Similarly, Domelier et al. (23) found that among 119 ERY-resistant *S. agalactiae* isolates, 55% of serotype Ia, and 75% of serotype Ib populations, harbored *mef*(A) and *erm*(A), respectively, whereas *erm*(B) was present in both serotype III (73%) and V isolates (75%).

Besides antibiotic selective pressure, the dominance of certain populations within a microbial species (bio- and/or genogroup) might depend on host specificity and virulence (31, 32). In *S. parauberis*, only serotypes I and II have been isolated from fish in South Korea and Japan (9, 10, 30). Conversely, those serotypes have never been isolated from turbot in Spain, which are known to be significantly more susceptible to *S. parauberis* serotype III (4, 29). Yolanda et al. (29) demonstrated that the lethal dose of 50% (LD_50_) of *S. parauberis* serotype III isolates for turbot was at least 10^5^ times lower than that of serotype I and II isolates, suggesting that *S. parauberis* might be a host-specific pathogen. Likewise, Han et al. (30) demonstrated that among serotypes of *S. parauberis* isolated from South Korea, serotype II was the least pathogenic to olive flounder, which might be associated with the very low frequency found in this study (~8%, 8/103).In this study, recent isolates belonging to serotype Ib/Ic and II tended to be classified as NWT with resistance against more antimicrobials, including AMX, OTC, and ERY, compared to the old isolates (Fig. 2 and Fig. S3). Those therapeutic agents are most commonly used in the field (33), and their frequent use might be associated with increased NWT populations with those serotypes. Buschmann et al. (34) showed a significant increase in the prevalence of ARGs in sediments from the vicinity of fish farms compared to those from control sites. Those results indicate that there is a clear correlation between the use of antibiotics and the prevalence of antibiotic resistance, as previously described for various bacterial species (35 – 37). While old serotype Ia isolates were commonly categorized as NWT with resistance to OTC and ERY, many recent serotype Ia isolates obtained from 2018 to 2021 (*n* = 28/45) were classified as NWT with resistance to only OTC, but not ERY, as they lacked *erm*(B) (Fig. 3 and 4B). Since *erm*(B) mediates resistance to both lincosamide and macrolide antibiotics (38), the rate of NWT populations with resistance to CLI was also significantly decreased (Fig. 2). Similarly, 19 serotype I isolates collected in the 2000s were commonly resistant to OTC and ERY (17), but 29 out of 56 serotype Ia isolates obtained in 2018–2019 were resistant only to OTC (18). Recent decreases in the number of serotype Ia isolates categorized as NWT with resistance to ERY might be due to reductions in ERY use by oral administration and increases in the use of amoxicillin by injection in the field, as shown in a study by Kim et al. (33).

Almost all serotype Ia isolates and about half of serotype Ib/Ic and II isolates were categorized as NWT populations with resistance to AMX, generally known to be effective for Gram-positive bacteria (39, 40). Resistance against AMX can be often introduced through point mutations in genes encoding for penicillin-binding proteins (PBPs) (41 – 43). As found by Woo et al. (18), it was presumed that the NWT populations of *S. parauberis* isolates used in this study were due to point mutations in *pbp1A* and *pbp2x*. Serotype Ia and II isolates classified as NWT with resistance to OTC and ERY harbored *tet*(S) and *erm*(B), and *tet*(M) and *mef*(J)-*msr*(I), respectively. These genes are responsible for resistance against OTC and ERY, respectively. Similarly, previous studies (17, 18) identified *tet*(S) and *erm*(B) in OTC and ERY-resistant populations of serotype Ia, respectively, and *tet*(M) in OTC-resistant serotype II. However, none of those studies identified the ARGs responsible for ERY resistance in serotype II. The genome-wide analysis in this study successfully identified the presence of an adjacent gene pair, *mef*(J)-*msr*(I) in *S. parauberis* serotype II genomes (Fig. 3 and 4B). Several variants of the *mef-msr* genes, which encode a dual-efflux system targeting macrolide antibiotics were previously reported (44, 45). In this study, *mef*(J) and *msr*(I) present in *S. paruaberis* serotype II showed ~98% and ~75% amino acid homology with *mef*(J)-*msr*(I) in *Streptococcus pyogenes* (Genbank accession number, QQA64075-6) and *mef*(D)-*msr*(F) in *Macrococcus canis* (Genbank accession number, QHW12307-8), respectively. Previous studies (46, 47) also showed that *mef* and *erm* mediated low- and high-level resistance to ERY, respectively, in both *S. pneumoniae* and *S. pyogenes*. It was also found that isolates harboring *mef*(J)-*msr*(I) showed low-level resistance against ERY, as shown in Fig. 4B.

BLAST search using the Comprehensive Antibiotic Resistance Database (48) indicated that all *S. parauberis* genomes used in this study harbored *mre*(A) and *pat*(A)-*pat*(B). Although *mre*(A) was previously identified as responsible for macrolide resistance in *S. agalactiae* (49), recent studies (50, 51) found that *mre*(A) was ubiquitous in *S. agalactiae* and was not directly related to macrolide resistance. The other genes, *pat*(A)-*pat*(B), are known to be ABC transporters that function as an efflux pump for various drugs, as well as toxic compounds in *S. pneumoniae* (52 – 54). However, *pat*(A)-*pat*(B) in *S. parauberis* shared only ~67% amino acid similarity with *pat*(A)*-pat*(B) in *S. pneumoniae* (Genbank accession numbers: AAK76136.1 and AAK76137.1) and were present in all *S. parauberis* genomes used in this study, indicating that these genes might be ubiquitous and not involved in drug resistance in *S. parauberis*. Hence, those two ARGs were excluded from the results since, as no evidence of antibiotic resistance could be found.

A study by Woo et al. (18), using 83 *S*. *parauberis* isolates derived from fish in South Korea, found that *tet*(S) and *tet*(M) were present in all OTC-resistant serotype Ia and II strains, respectively, which was in line with results of this study (Fig. 4A). However, Woo et al. (18) also reported that 5%, ~43%, and ~17% of the OTC-resistant serotype Ia isolates possessed *tet*(A), *tet*(B), and *tet*(Y), respectively, and ~67% of OTC resistant serotype II harbor *tet*(B). Some of the OTC-resistant isolates contained more than one tetracycline resistance genes, but we could not specify the exact proportion of those populations from the results presented by Woo et al. (18). We have not identified *tet*(A/B/Y) either by analysis of publicly available *S. parauberis* genome sequences and PCR tests using specific primer sets targeting those genes (data not shown). In general, tetracycline resistance in Streptococci is induced through an efflux pump encoded by the *tet*(K/L) gene and ribosomal protection mediated by *tet*(M/O/Q/S/T/W) (43). In fact, *tet*(A/B/Y) are frequently found in Gram-negative bacteria, whereas they are extremely rare (55, 56) or have never been reported (in case of *tet*(Y)) in other Streptococci.

## MATERIALS AND METHODS

### Bacterial isolation, identification, and serotyping

This study used a total of 103 strains, isolated from 1999 to 2021 from olive flounder and starry flounder and were identified as *S. parauberis* using the method previously described (57) (Table S1). Bacteria were isolated from swabs of the liver, kidney, and/or spleen of diseased fish by inoculating the swabs on brain heart infusion (BHI) agar plates supplemented with 1% NaCl (BN; Oxoid, UK). The plates were incubated at 28°C for 24–48 h. The bacterial isolates were pure-cultured on the same media and identified as *S. parauberis* using the PCR method developed by Mata et al. (58). For bacterial serotyping, a multiplex PCR assay developed by Tu et al. (9) was employed. Accordingly, the serotypes of *S. parauberis* isolates could be determined as Ia, Ib/Ic, and II types. Briefly, genomic DNA was extracted from the isolates using an AccuPrep Genomic DNA Extraction Kit (Bioneer, South Korea) and PCR was conducted using AccuPower PCR PreMix (Bioneer, South Korea) as described in previous studies (9, 58). The primer list is summarized in Table S3. The isolates were stored at −80°C in BHI broth supplemented with 10% glycerol.

### Antimicrobial susceptibility testing

The drug susceptibility of *S. parauberis* isolates was determined by the disk diffusion test according to CLSI guideline VET03-A (27). Briefly, overnight cultured bacteria were adjusted to a concentration of 2 × 10^8^ CFU mL^−1^ and inoculated onto Mueller-Hinton agar containing 5% sheep blood using a sterile swab. The following commercial antibiotic disks (Oxoid) were placed on the lawn culture: amoxicillin 25 µg, oxytetracycline 30 µg, cephalexin 30 µg, ceftiofur 30 µg, enrofloxacin 5 µg, erythromycin 15 µg, sulfamethoxazole (23.75 µg)/trimethoprim (1.25 µg) 25 µg, clindamycin 10 µg, florfenicol 30 µg, and chloramphenicol 30 µg. *Escherichia coli* ATCC 25922 and *Aeromonas salmonicida* ATCC 33658 were used as the reference strains for quality control (27).

### Data analysis

In this study, the ECV was determined through the NRI method developed by Kronvall and Smith (59). Mean values and standard deviations of WT normalized zone sizes were calculated using a plot of the probit values of the normalized accumulative frequencies of observations against zone diameters (59). The ECV was set at two and a half standard deviations below the mean (59). Principal component analysis and hierarchical clustering analysis were performed based on bacterial antibiotic susceptibility patterns using ClustVis (60) and Morpheus (https://software.broadinstitute.org/morpheus), respectively. Significant differences among groups were analyzed using the Kruskal–Wallis test, followed by the post hoc Dunn’s test with Bonferroni correction (significance at adjusted *P* values <0.05) using SPSS v23.0 (IBM, USA).

### Identification of ARGs in whole-genomic sequences of *S. parauberis*

In addition to the four genome sequences obtained from this study (see supplementary results), 27 *S*. *parauberis* genomic sequences were obtained from the National Biotechnology Information Center (NCBI; https://www.ncbi.nlm.nih.gov/data-hub/genome/?taxon=1348) and used for the analysis (Table S4). The serotypes of a total of 31 strains were determined through *in silico* PCR (Primer-BLAST) using the primers listed in Table S3 (61). ARGs in the genomic sequences were identified using Resistance Gene Identifier in the Comprehensive Antibiotic Resistance Database (48) and illustrated using EasyFig v2.2.2 (62).

### Detection of ARGs in *S. parauberis* isolates

Genomic DNA extracted from 103 *S*. *parauberis* strains was used for the identification of ARGs. PCR was conducted to evaluate the presence of three genes, *erm*(B), *mef*(J), and *msr*(I), involved in macrolide resistance, and two tetracycline resistance genes, *tet*(M) and *tet*(S). The primers used in this study are listed in Table S3. PCR was conducted as described in previous studies (63 – 65). In this study, primer sets for *mef*(J) and *msr*(I) were designed using Primer3Plus (66), based on coding DNA sequences retrieved from the whole-genomic sequence of *S. paruaberis* KSP10 (Table S4). The PCR conditions were as follows: pre-denaturation at 95°C for 5 min, 30 cycles of denaturation at 95°C for 30 s, annealing at 55°C for 30 s, extension at 72°C for 30 s, and a final extension at 72°C for 7 min. PCR products were identified by electrophoresis in 1.5% (wt/vol) agarose gel stained with ethidium bromide.

## Data Availability

The complete genome sequences in this project have been deposited in BioProject accession number PRJNA940317. All data that support the findings of this study are available on reasonable request to the corresponding author.

## SUPPLEMENTAL MATERIAL

Supplemental materials spectrum.04400-22-s0001.pdf This file includes supplementary materials and methods, supplementary results, Tables S1 to S4, Figures S1 to S3, and references.

ASM does not own the copyrights to Supplemental Material that may be linked to, or accessed through, an article. The authors have granted ASM a non-exclusive, world-wide license to publish the Supplemental Material files. Please contact the corresponding author directly for reuse.
