# Supplementary material for: Serotype distribution and antibiogram of Streptococcus parauberis isolated from fish in South Korea
Source: Microbiol Spectr. 2023 Aug 9;11(5):e04400-22. doi: 10.1128/spectrum.04400-22 (PMC10581249; doi:10.1128/spectrum.04400-22)
Supplement: Supplemental materials — This file includes supplementary materials and methods, supplementary results, Tables S1 to S4, Figures S1 to S3, and references. [file spectrum.04400-22-s0001.pdf]

Supplementary materials and methods

Whole genome sequencing

In this study, whole genome of four *S. parauberis* isolates (KSP45, SPOF19J15, SPOF21J2 and SPOF21J26) were sequenced based on Oxford Nanopore sequencing platform as previously described with slight modifications (**1**). Genomic DNA were extracted using the Wizard genomic DNA purification kit (Promega, USA) and quantified using a Qubit 3.0 fluorometer (Invitrogen, USA) and a Qubit double-stranded DNA (dsDNA) high-sensitivity (HS) kit (Invitrogen) according to the manufacturers' instructions. Sequencing libraries were prepared using the ligation sequencing kit (SQK-LSK109; Oxford Nanopore Technologies [ONT], UK) with the barcoding kit (EXP-NBD104; ONT), following the manufacturers' instructions. Whole genome sequencing was performed on MinION Mk1B system (ONT) using a SpotON R9.4.1 flow cell (ONT). Base-calling was performed using MinKNOW v19.10.1 software (ONT) with a high-accuracy base-calling mode. After assessment of sequencing quality using MinIONQC v1.4.2 (**2**), *de novo* assembly and polishing were performed using Flye v2.9 (**3**) and Medaka v.1.2.1 (https://github.com/nanoporetech/medaka). The quality of assembly was evaluated with QUAST v5.0.2 (**4**). Genes were predicted and annotated based on the National Center for Biotechnology Information (NCBI) Prokaryotic Genome Annotation Pipeline (PGAP) v6.2 (**5**).

Supplementary results

Whole genome sequencing

In this study, four *S. parauberis* genomes (KSP45 and SPOF19J26, serotype Ia; SPOF19J15, serotype Ib/Ic; SPOF21J2, serotype II) were sequenced based on Oxford Nanopore sequencing platform. Briefly, an average of 27,845 Nanopore long reads (an average N_{50}, 18,092 bp) were generated, which were then assembled in complete level (an average sequencing depth, 247x). Briefly, genomes of KSP45, SPOF19J15 and SPOF21J2 were composed of a single circular chromosome with size of 2.15, 1.99 and 2 Mb, respectively. SPOF21J26 harbored an ~12 kb-sized plasmid, in addition to 2.07 Mb-sized chromosome, which could be also found in genome sequences of other serotype Ia genomes as previously demonstrated (**6, 7**). More detailed information on genome is available in Table S4 and BioProject accession PRJNA940317.

Supplementary tables

Table S1. Year of isolation, source, origin, and serotype of *Streptococcus parauberis* strains used in this study

No.	Strain code	Year of isolation	Origin	Source (Province)	Serotype
1	KSP44	1999	Olive flounder	Jeju Island	Ia
2	KSP45	1999	Olive flounder	Jeju Island	Ia
3	KSP46	1999	Olive flounder	Jeju Island	Ia
4	KSP47	1999	Olive flounder	Gyeongsangbukdo (Gyeongsang)	Ia
5	KSP1	2003	Olive flounder	Jeju Island	II
6	KSP2	2003	Olive flounder	Jeju Island	Ia
7	KSP3	2003	Olive flounder	Jeju Island	Ia
8	KSP4	2003	Olive flounder	Jeju Island	II
9	KSP5	2004	Olive flounder	Jeju Island	Ib/Ic
10	KSP6	2004	Olive flounder	Jeju Island	Ib/Ic
11	KSP7	2004	Olive flounder	Jeju Island	Ia
12	KSP8	2004	Olive flounder	Jeju Island	Ia
13	KSP9	2004	Olive flounder	Jeju Island	Ia
14	KSP10	2004	Olive flounder	Jeju Island	II
15	KSP12	2005	Olive flounder	Jeju Island	Ib/Ic
16	KSP13	2005	Olive flounder	Jeju Island	Ib/Ic
17	KSP14	2005	Olive flounder	Jeju Island	II
18	KSP15	2005	Olive flounder	Jeju Island	Ib/Ic
19	KSP16	2005	Olive flounder	Jeju Island	Ib/Ic
20	KSP17	2005	Olive flounder	Jeju Island	Ib/Ic
21	KSP20	2005	Olive flounder	Jeju Island	Ib/Ic
22	KSP22	2005	Olive flounder	Haenam (Jeolla)	Ib/Ic
23	KSP23	2005	Olive flounder	Haenam (Jeolla)	Ib/Ic
24	KSP24	2005	Olive flounder	Wando (Jeolla)	Ib/Ic
25	KSP26	2005	Olive flounder	Wando (Jeolla)	Ib/Ic
26	KSP27	2005	Olive flounder	Wando (Jeolla)	Ib/Ic
27	KSP28	2005	Olive flounder	Wando (Jeolla)	Ib/Ic
28	KSP34	2005	Olive flounder	Wando (Jeolla)	Ib/Ic
29	KSP38	2005	Olive flounder	Wando (Jeolla)	Ib/Ic
30	PH0710	2007	Starry flounder	Pohang (Gyeongsang)	Ia
31	PH0711	2007	Starry flounder	Pohang (Gyeongsang)	Ia
32	SPOF3K	2013	Olive flounder	Geojedo (Gyeongsang)	Ia
33	04151K	2013	Olive flounder	Geojedo (Gyeongsang)	Ia
34	04162K	2013	Olive flounder	Geojedo (Gyeongsang)	Ia
35	HFTC0023	2014	Olive flounder	Pohang (Gyeongsang)	Ia
36	HFTC0059	2014	Olive flounder	Pohang (Gyeongsang)	Ia
37	HFTC0060	2014	Olive flounder	Pohang (Gyeongsang)	Ia
38	HFTC0063	2014	Olive flounder	Pohang (Gyeongsang)	Ia
39	HFTC0064	2014	Olive flounder	Pohang (Gyeongsang)	Ia
40	HFTC0076	2014	Starry flounder	Yeongdeok (Gyeongsang)	Ib/Ic
41	HFTC0083	2015	Starry flounder	Yeongdeok (Gyeongsang)	Ia
42	HFTC0086	2015	Starry flounder	Uljin (Gyeongsang)	Ia
43	HFTC0091	2015	Starry flounder	Uljin (Gyeongsang)	Ia
44	HFTC0157	2015	Starry flounder	Pohang (Gyeongsang)	Ia
45	HFTC0237	2015	Starry flounder	Yeongdeok (Gyeongsang)	Ia
46	SPOF18J1	2018	Olive flounder	Jeju Island	Ia
47	SPOF18J2	2018	Olive flounder	Jeju Island	Ia

48	SPOF18J3	2018	Olive flounder	Jeju Island	Ia
49	SPOF18J4	2018	Olive flounder	Jeju Island	Ia
50	SPOF18J5	2018	Olive flounder	Jeju Island	Ia
51	SPOF18J6	2018	Olive flounder	Jeju Island	Ib/Ic
52	SPOF18J7	2018	Olive flounder	Jeju Island	Ib/Ic
53	SPOF18J8	2018	Olive flounder	Jeju Island	Ia
54	SPOF18J9	2018	Olive flounder	Jeju Island	Ia
55	SPOF18J10	2018	Olive flounder	Jeju Island	Ia
56	SPOF18J11	2018	Olive flounder	Jeju Island	Ib/Ic
57	SPOF18J12	2018	Olive flounder	Jeju Island	Ia
58	SPOF18J13	2018	Olive flounder	Jeju Island	Ib/Ic
59	SPOF18G1	2018	Starry flounder	Uljin (Gyeongsang)	Ia
60	SPOF18G2	2018	Starry flounder	Goseong (Gyeongsang)	Ia
61	SPOF19J1	2019	Olive flounder	Jeju Island	Ib/Ic
62	SPOF19J2	2019	Olive flounder	Jeju Island	Ib/Ic
63	SPOF19J3	2019	Olive flounder	Jeju Island	Ia
64	SPOF19J4	2019	Olive flounder	Jeju Island	Ia
65	SPOF19J5	2019	Olive flounder	Jeju Island	Ia
66	SPOF19J6	2019	Olive flounder	Jeju Island	Ib/Ic
67	SPOF19J7	2019	Olive flounder	Jeju Island	Ia
68	SPOF19J8	2019	Olive flounder	Jeju Island	Ia
69	SPOF19J9	2019	Olive flounder	Jeju Island	Ia
70	SPOF19J10	2019	Olive flounder	Jeju Island	Ia
71	SPOF19J11	2019	Olive flounder	Jeju Island	Ia
72	SPOF19J12	2019	Olive flounder	Jeju Island	Ia
73	SPOF19J13	2019	Olive flounder	Jeju Island	Ia
74	SPOF19J14	2019	Olive flounder	Jeju Island	Ia
75	SPOF19J15	2019	Olive flounder	Jeju Island	Ib/Ic
76	SPOF19P1	2019	Olive flounder	Pohang (Gyeongsang)	Ia
77	SPOF21J1	2021	Olive flounder	Jeju Island	Ia
78	SPOF21J2	2021	Olive flounder	Jeju Island	II
79	SPOF21J3	2021	Olive flounder	Jeju Island	Ia
80	SPOF21J4	2021	Olive flounder	Jeju Island	II
81	SPOF21J5	2021	Olive flounder	Jeju Island	Ia
82	SPOF21J6	2021	Olive flounder	Jeju Island	Ia
83	SPOF21J7	2021	Olive flounder	Jeju Island	Ia
84	SPOF21J8	2021	Olive flounder	Jeju Island	Ia
85	SPOF21J9	2021	Olive flounder	Jeju Island	Ia
86	SPOF21J10	2021	Olive flounder	Jeju Island	Ia
87	SPOF21J11	2021	Olive flounder	Jeju Island	Ia
88	SPOF21J12	2021	Olive flounder	Jeju Island	Ia
89	SPOF21J13	2021	Olive flounder	Jeju Island	Ia
90	SPOF21J14	2021	Olive flounder	Jeju Island	Ia
91	SPOF21J15	2021	Olive flounder	Jeju Island	Ia
92	SPOF21J16	2021	Olive flounder	Jeju Island	II
93	SPOF21J17	2021	Olive flounder	Jeju Island	Ia
94	SPOF21J18	2021	Olive flounder	Jeju Island	Ia
95	SPOF21J19	2021	Olive flounder	Jeju Island	Ia
96	SPOF21J20	2021	Olive flounder	Jeju Island	II
97	SPOF21J21	2021	Olive flounder	Jeju Island	Ia
98	SPOF21J22	2021	Olive flounder	Jeju Island	Ia
99	SPOF21J23	2021	Olive flounder	Jeju Island	Ia
100	SPOF21J24	2021	Olive flounder	Jeju Island	Ia
101	SPOF21J25	2021	Olive flounder	Jeju Island	Ia
102	SPSF21J1	2021	Starry flounder	Jeju Island	Ib/Ic
103	SPOF21J26	2021	Olive flounder	Jeju Island	Ia

Table S2. Antibiotic disk zone diameters of 103 strains of *Streptococcus parauberis*

Zone (mm)	Antibiotics*									
	AMX	OTC	ERY	FFC	ENR	SXT	CFL	EFT	CLI	CHL
6		68	29						3	
7		1	2							
8		1	1							
9		2	2							
10		1	4							
11		1	1							
12		1							1	
13		1							3	
14							3	1	3	
15							2		1	
16							1		4	
17	1					1	2		3	
18			1		1	3	1		2	
19			1			2			8	
20	2		3		1	2	2			
21	4		2	1	1	9	3	3	2	
22	3	1			4	9	3	1	1	
23	6	2	1	1	5	12	2	4	2	
24	14			1	6	14	8	4	1	
25	15			2	10	14	13	3	1	2
26	10	2	1	4	15	7	12	7		10
27	4	1		3	11	16	9	1	1	11
28	8	1	1	8	12	7	6	4		12
29	4	2		7	8	4	10	6	6	20
30	4	1	15	17	14	3	4	17	14	16
31	5	5	6	18	5		1	5	12	3
32	3	4	13	22	7		4	17	13	13
33		2	1	4	1		2	2	5	4
34		3	3	8				5	8	6
35		1	4	5			2	4	2	3
36	3	2	4		1		3	3	4	2
37			3	1			3	2	2	1
38	3		2	1			2	6		
39	2		2		1			4		
40	4						5	1	1	
41	7		1					1		
42								2		
43	1									

*Abbreviations for antibiotic agent are as in the materials and methods.

**Areas in grey indicate the range of zones for wild-type isolates.

Table S3. Primers used in this study

Primer*	Sequences (5’-3’)	Product size	Reference
**Identification**			
Spa-2152 -F	TTTCGTCTGAGGCAATGTTG	718	8
Spa-2870 -R	GCTTCATATATCGCTATACT		
**Serotyping**			
Serotype Ia -F	ATTGTTAGTCATTCAGTTGT	213	9
Serotype Ia -R	AATTATAGTCAACAGTCCAG		
Serotype Ib/Ic -F	ATTTCTACCAGGTTACTTTG	303	9
Serotype Ib/Ic -R	ACATCTCGAAACTTCATATT		
Serotype II -F	GAACTACTTAGGTTTAGCAT	413	9
Serotype II -R	AACTTGTAAATAGGATTGCT		
Serotype III -F	GACCAACACCAGCACCAATA	130	10
Serotype III -R	TTTGGACAACCTGGAAGAGC		
**Antibiotic resistance gene**			
tet(M) -F	GTTAAATAGTGTTCTTGGAG	657	11
tet(M) -R	CTAAGATATGGCTCTAACAA		
tet(S) -F	CATAGACAAGCCGTTGACC	667	12
tet(S) -R	ATGTTTTTGGAACGCCAGAG		
erm(B) -F	GTAAACAGTTGACGATATTCTCG	224	13
erm(B) -R	CGTACCTTGGATATTCACCG		
mef(J) -F	CTTTTGCTGGCACGATTACA	182	This study
mef(J) -R	TGCTGGCATATGAAAAGCAG		
msr(I) -F	TTCGAAAGGGCTAAGTGGTG	229	This study
msr(I) -R	CCCATCATCAACTTCCCAAA		

*F, forward; R, reverse.

Table S4. General features of *Streptococcus parauberis* whole genome sequences used in this study

Strain	Level	Genome size (Mbp)	No. of CDS	Serotype	Origin	Region of isolation	Year of isolation	Accession number	Reference
**SPOF21J26**	Complete	2.08	1,827	Ia	Olive flounder	South Korea	2021	CP119428-9	This study
**SPOF19J15**	Complete	1.99	1,736	II	Olive flounder	South Korea	2019	CP119426	This study
**SPOF21J2**	Complete	2	1,736	Ib/Ic	Olive flounder	South Korea	2021	CP119430	This study
**KSP45**	Complete	2.15	2,016	Ia	Olive flounder	South Korea	1999	CP119427	This study
**SPOF3K**	Complete	2.15	2,049	Ia	Olive flounder	South Korea	2013	CP025420-1	6
**HFTC0064**	Complete	2.16	2,155	Ia	Olive flounder	South Korea	2014	CP104190-1	7
**KSP20**	Complete	2.23	2,255	Ib/Ic	Olive flounder	South Korea	2005	CP104048	7
**KSP10**	Complete	2.08	2,020	II	Olive flounder	South Korea	2004	CP104047	7
**KSP14**	Complete	2.08	2,020	II	Olive flounder	South Korea	2005	CP104046	7
**KCTC 11537**	Complete	2.14	1,868	Ib/Ic	Olive flounder	South Korea	2006	CP002471	14
**KCTC 11980BP**	Scaffold	2.14	1,890	Ia	Olive flounder	South Korea	2010	BANN01	15
**KRS-02083**	Contig	2.18	2,143	Ia	Olive flounder	Japan	2002	ALYM01	14
**KRS-02109**	Contig	2.05	1,966	II	Olive flounder	Japan	2002	ALWR01	14
**SK-417**	Contig	1.96	1,871	Ib/Ic	Black seaperch	Japan	2013	BAWT01	16
**T1**	Contig	2.01	1,909	III	Turbot	Spain	1997	LQRN01	10
**AZ70.1**	Contig	2.02	1,907	III	Turbot	Spain	1994	NSGS01	10
**N11**	Contig	2	1,976	III	Striped bass	USA	2002	LIXR01	17
**PL23**	Contig	2.02	1,985	III	Striped bass	USA	2002	LHAD01	17
**PL9**	Contig	2.01	1,993	III	Striped bass	USA	2002	LJCT01	17
**RP15**	Contig	2.01	1,959	III	Striped bass	USA	2002	LRBI01	17
**RP17**	Contig	2.02	1,964	III	Striped bass	USA	2002	LRBJ01	17
**RP25**	Contig	2.03	1,953	III	Striped bass	USA	2002	LRBK01	17
**N198_2**	Contig	1.98	1,924	III	Striped bass	USA	2006	LHAC01	17
**FSL S3-0563**	Contig	2.13	2,059	Others	Cow (milk)	USA	2001	LHAE01	NA*
**FSL R5-0305**	Contig	2.09	1,939	Others	Cow (milk)	USA	2006	LRFJ01	NA
**FSL R5-303**	Contig	2.08	1,956	Others	Cow (milk)	USA	2006	LRDN01	NA
**FSL W6-0838**	Contig	2.11	1,987	Others	Cow (milk)	USA	NA	LRBL01	NA
**SP204-42-2**	Contig	2.13	2,010	Others	Cow (milk)	Finland	2001	LQRM01	NA
**SP158-29**	Contig	2.09	2,021	Others	Cow (mastitis)	Finland	2001	NSGR01	NA
**NCFD 2020**	Chromosome	2.16	2,059	Others	Cow (mastitis)	UK	NA	AEUT02	NA
**Fin349**	Contig	2.18	2,056	Others	Packaged chicken	Finland	2001	LQRO01	NA

* NA, Not available.

Supplementary Figures

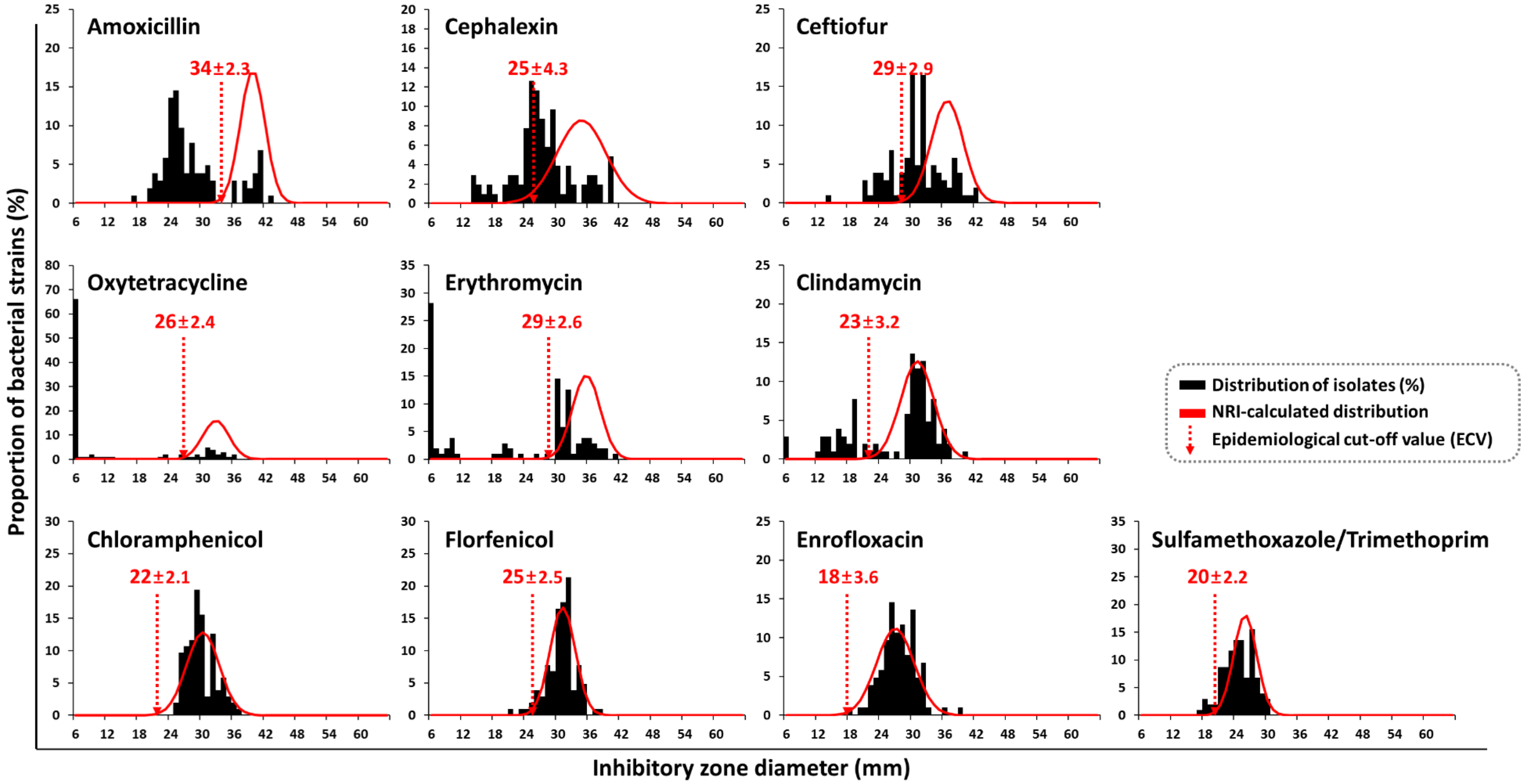

Figure S1. Inhibitory zone diameter histogram of disk diffusion tests of ten antibiotics in 103 *Streptococcus parauberis* isolates from fish. The normalized resistance interpretation (NRI)-calculated theoretical populations of wild type strains are shown as red line graphs, and the epidemiological cut-off values (ECV) are shown above the red arrows with standard deviations.

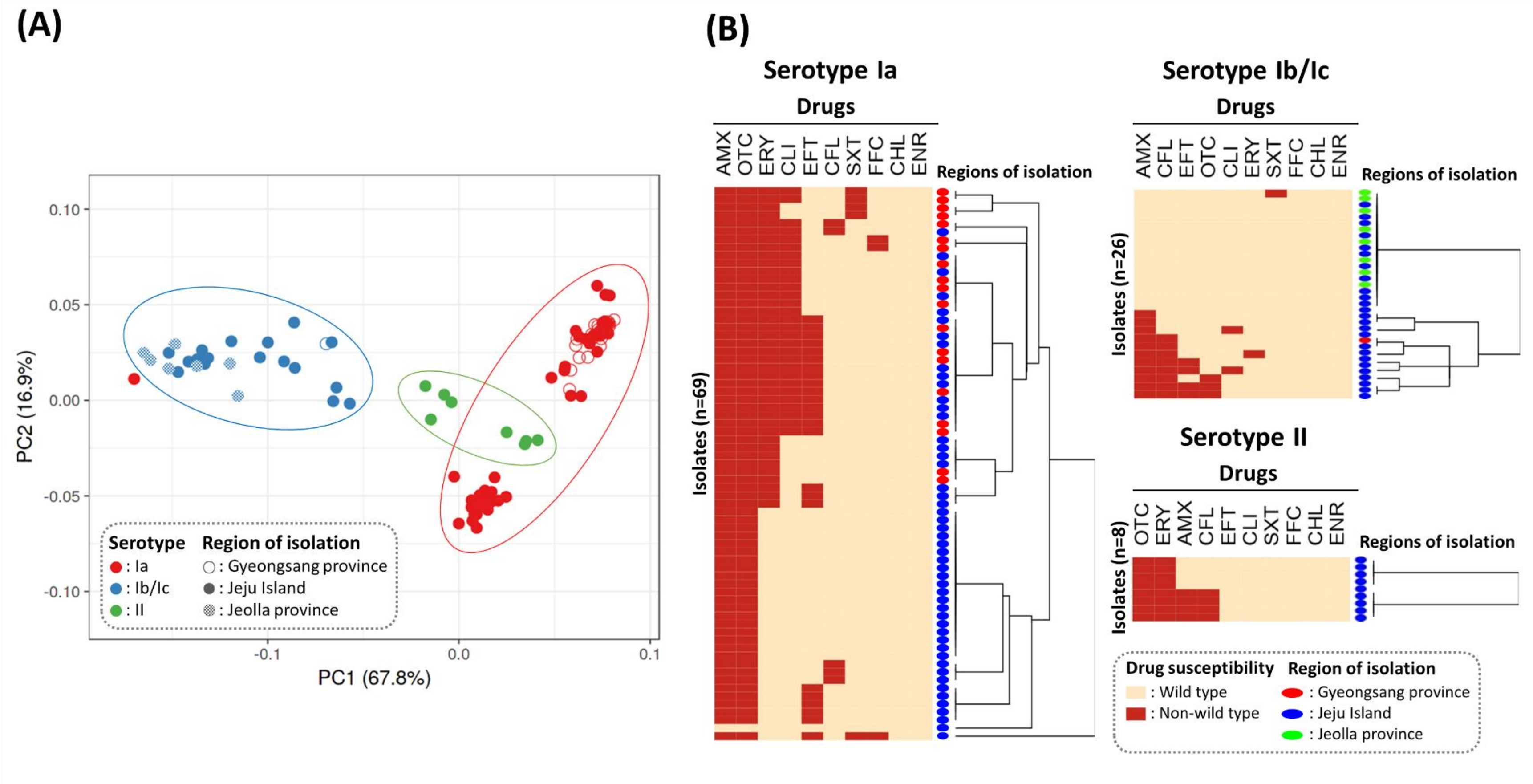

Figure S2. Principal component analysis (PCA; A) and hierarchical clustering analysis (B) based on the antibiogram of 103 *Streptococcus parauberis* isolates against ten antibiotics. (A) The PCA plot captures the variance in the dataset composed of normalized inhibitory zone diameters obtained from the disk diffusion tests of 103 *S. parauberis* strains. The X and Y-axes show principal components 1 and 2, which explain 67.8% and 16.9% of the total variance, respectively. The color and shape of the marks indicate serotypes and the region of isolation. (B) Heatmaps show the wild type (beige) and non-wild type (red) distributions of *S. parauberis* isolates according to serotype. The red, blue, and green ellipses, located on the right side of the heatmap indicate the region of isolation. Abbreviations for the antibiotic agents are shown in Table 2.

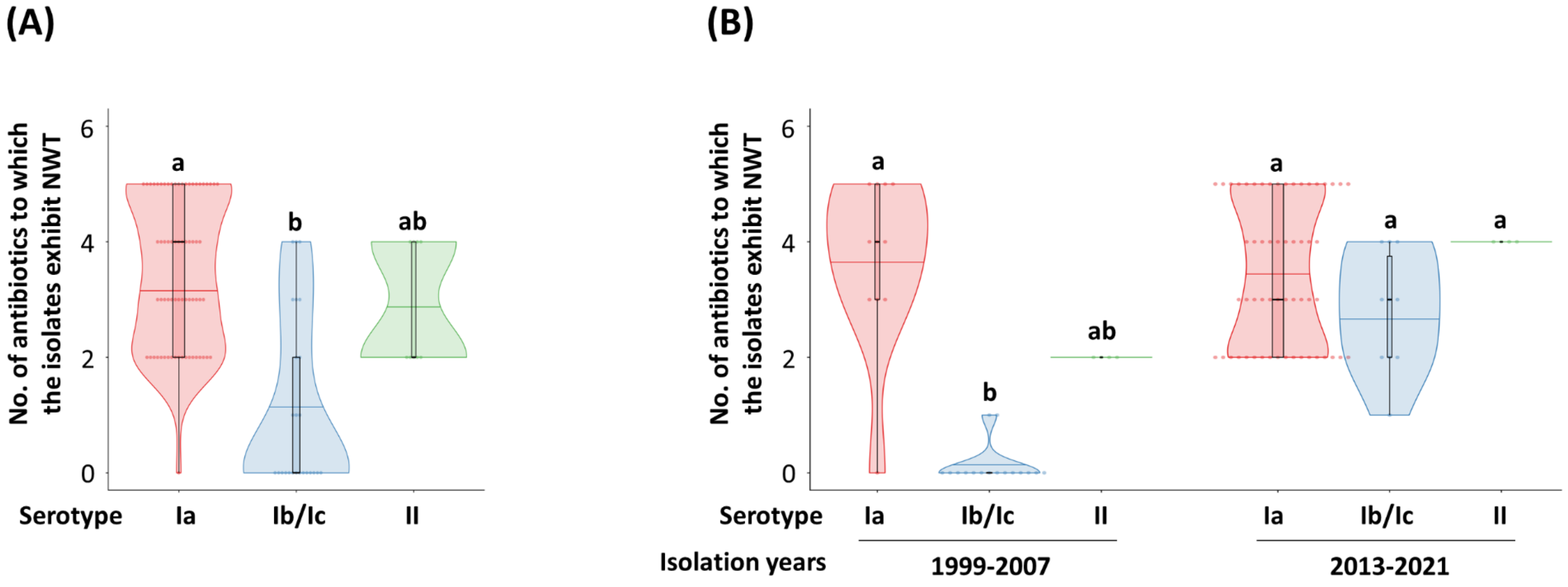

Figure S3. Violin plot demonstrating the number of antibiotics to which the isolates exhibit non-wild type (NWT) in *Streptococcus parauberis*. The number of NWT-classified antibiotics in respective isolates are presented as dots inside the violin plot and box plot. The violin plot represents the minimum value to maximum value with the probability density of the data. In the box plot, the bold line inside the box, box boundaries, and error lines represent the median value, 25th – 75th and 10th – 90th percentiles, respectively. The width of the box indicates the relative number of samples. Statistical differences were analyzed using the Kruskal–Wallis test, followed by the post hoc Dunn's test with Bonferroni correction (significance at adjusted p-value < 0.05).

References

1. Kim N, Lee Y, Tho HD, Park JY, Lee JY, Kang Y, Kim D. 2022. Whole-Genome Sequences of Three Edwardsiella piscicida Isolates from Diseased Fish in South Korea. Microbiology Resource Announcements **11**:e00097-22.

2. Lanfear R, Schalamun M, Kainer D, Wang W, Schwessinger B. 2019. MinIONQC: fast and simple quality control for MinION sequencing data. Bioinformatics **35**:523–525.

3. Kolmogorov M, Yuan J, Lin Y, Pevzner PA. 2019. Assembly of long, error-prone reads using repeat graphs. Nat Biotechnol **37**:540–546.

4. Mikheenko A, Prjibelski A, Saveliev V, Antipov D, Gurevich A. 2018. Versatile genome assembly evaluation with QUAST-LG. Bioinformatics **34**:i142–i150.

5. Tatusova T, DiCuccio M, Badretdin A, Chetvernin V, Nawrocki EP, Zaslavsky L, Lomsadze A, Pruitt KD, Borodovsky M, Ostell J. 2016. NCBI Prokaryotic Genome Annotation Pipeline. Nucleic Acids Res **44**:6614–6624.

6. Lee Y, Nguyen TL, Kim A, Kim N, Roh HJ, Han H, Jung S, Cho M, Kang HY, Kim D. 2018. Complete genome sequence of multiple-antibiotic-resistant Streptococcus parauberis strain SPOF3K, isolated from diseased olive flounder (Paralichthys olivaceus). Genome announcements **6:**e00248-18.

7. Lee Y, Kim A, Kim N, Roh H, Ho DT, Kim D. 2023. Complete Genome Sequences of Streptococcus parauberis Serotype Ia, Ib/Ic, and II Isolates from Diseased Olive Flounder in South Korea. Microbiology Resource Announcements e01058-22.

8. Mata AI, Gibello A, Casamayor A, Blanco MM, Domínguez L, Fernández-Garayzábal J. 2004. Multiplex PCR assay for detection of bacterial pathogens associated with warm-water streptococcosis in fish. Appl Environ Microbiol **70:**3183-3187.

9. Tu C, Suga K, Kanai K. 2015. A Multiplex PCR Assay for Different-iation of Streptococcus parauberis Serotypes. Fish Pathol **50:**213-215.

10. Torres-Corral Y, Santos Y. 2020. Comparative genomics of Streptococcus parauberis: new target for molecular identification of serotype III. Appl Microbiol Biotechnol **104:**6211-6222.

11. Aarestrup FM, Agerso Y, Gerner–Smidt P, Madsen M, Jensen LB. 2000. Comparison of antimicrobial resistance phenotypes and resistance genes in Enterococcus faecalis and Enterococcus faecium from humans in the community, broilers, and pigs in Denmark. Diagn Microbiol Infect Dis **37:**127-137.

12. Ng L, Martin I, Alfa M, Mulvey M. 2001. Multiplex PCR for the detection of tetracycline resistant genes. Mol Cell Probes **15:**209-215.

13. Nagai K, Shibasaki Y, Hasegawa K, Davies TA, Jacobs MR, Ubukata K, Appelbaum PC. 2001. Evaluation of PCR primers to screen for Streptococcus pneumoniae isolates and β-

lactam resistance, and to detect common macrolide resistance determinants. J Antimicrob Chemother **48:**915-918.

14. Nho S, Hikima J, Park SB, Jang HB, Cha IS, Yasuike M, Nakamura Y, Fujiwara A, Sano M, Kanai K. 2013. Comparative genomic characterization of three Streptococcus parauberis strains in fish pathogen, as assessed by wide-genome analyses. PLoS One **8:**e80395.

15. Park MA, Kwon MG, Hwang JY, Jung SH, Kim D, Park J, Kim J, Na Y, Kim M, Kim D. 2013. Genome sequence of Streptococcus parauberis strain KCTC11980, isolated from diseased Paralichthys olivaceus. Genome announcements **1:**e00780-13.

16. Oguro K, Yamane J, Yamamoto T, Ohnishi K, Oshima S, Imajoh M. 2014. Draft genome sequence of Streptococcus parauberis strain SK-417, isolated from diseased Sebastes ventricosus in Kagoshima, Japan. Genome announcements **2:**e00453-14.

17. Haines A, Nebergall E, Besong E, Council K, Lambert O, Gauthier D. 2016. Draft genome sequences for seven Streptococcus parauberis isolates from wild fish in the Chesapeake Bay. Genome Announcements **4:**e00741-16
